# Modern Sociology

**Published:** 1896-12-26

**Authors:** 


### Modern Sociology.

### THE WINNING OF GOLD.

So widespread is the recognition of gold as the type of riches, and so universal is its acceptance as the standard by which the value of all man's possessions shall be estimated, that a few lines on the modes in which this alluring metal is won from Mother Earth may be considered pertinent to the subject of sociology.

While most metals are dug from the earth as ores from which only by smelting and various metallurgical processes can the pure elements be obtained, gold is mostly found virgin and uncorrupt, either in the form of scales and tiny masses firmly held by the quartz and other rocks among which it lies, or as dust and grains of various sizes lying amid the sand which has resulted from the weathering of the rocks which formed its primæval bed.

From the earliest times gold has been collected from these sands by methods which are strictly analogous with those in use even up to the present day; and in almost every age, when the sands and gravels in which it had first been found became exhausted, attempts have been made to break down the rocks themselves, and extract the precious metal from its original matrix, and it is in this direction that the greatest advances have been made in recent times.

In *Science Progress* for August there is an interesting account of the various means adopted for winning gold. The simple and barbaric plan consisted in simple washing, for gold is so much heavier than the rocks with which it is associated that it sinks in water much more readily than they do. The miner's pan, a flat-bottomed iron vessel, with sloping sides, first used by the Californian pioneers, has now become the favourite implement for prospecting in all parts of the world; but on the larger scale the machines consist of slightly inclined troughs, through which streams of water are made to carry the auriferous sand, the gold being deposited and the sandy particles washed away. But here we come upon an additional means for the more easy and complete extraction of the metal, viz., amalgamation. If the gold always lay absolutely detached from its rocky bed, and always occurred in grains of appreciable size, mere washing would extract the great mass of it from the sand with which it is mixed; but the metal is very apt to be adherent to little particles of quartz which, by their lightness, prevent it from sinking, and a very large amount of gold occurs in a powder so exceedingly fine that it is carried away by the current.

Gold, however, is miscible with mercury in all proportions. Directly a particle of it is touched by a globule of mercury it is taken up into the mass, and although the amalgam thus formed is much less heavy than the gold itself, it is far more easily collected owing to the tendency which even the finest particles of mercury display to run together and form large globules.

In treating gold-bearing gravel on the large scale, mercury is sprinkled on while the water washes it along the trough, and the little globules rolling about collect the very finest particles of gold. The amalgam so produced is caught in "riffles," and obstructions of various sorts arranged along the bottom for the purpose, and after having the excess of mercury filtered out by being squeezed through bags of canvas or leather, the pasty amalgam which is left is placed in a retort, and the mercury driven off by heat.

In modern mining practice in the recovery of gold from quartz it is usual to apply the mercury while the rock is being pulverised by the stampers. Rock, water, and mercury are pounded together by stamps of enormous weight, and thus the mercury has full opportunity of coming in contact with every particle of gold which is open to its influence. In the recovery of the amalgam thus produced advantage is taken of its tendency to adhere to any amalgamated surface. Instead of being caught by "riffles" and blankets and hides, as in former times, the product of the crushing mill is made to pass in thin streams over and between amalgamated copper plates, to which the gold amalgam at once adheres. Even the most perfect amalgamation process, however, fails to extract all the gold, for much of it exists in minute particles within the substance of even the finest grains of quartz, and even in finer particles still when mixed with pyrites and other complex minerals. No doubt by prolonged grinding with mercury a good deal of this can be extracted, but it is a slow and expensive process.

In such cases as these it is common to roast the ore and then apply chlorine solution, by which the gold is slowly dissolved out. Unfortunately, gold is apt to occur in parts of the world where coal is far from easy to obtain, which renders the process an expensive one.

It is found, however, that gold is soluble in very dilute solutions of cyanide of potassium, and the introduction of these solutions for "leaching" is said to be "probably the most important event in the history of the metallurgy of gold since the first application of mercury to gold extraction."

The solutions used are very dilute, the favourite "strong" solution containing only from 0·25 to 0·30 per cent. of cyanide, while solutions containing only 0·01 per cent. are found to be efficacious if a somewhat longer time is allowed. When the gold has been dissolved out it can be recovered from the cyanide by precipitation on zinc or by electrolysis.

At the present time the annual production of gold by these various methods is said to amount to about £42,000,000, which is greater than at any previous period in history, and double the output of seven years ago.

Of this total, washing processes are probably responsible for no more than 30 per cent. The amalgamation of crushed vein stuff produces about 55 per cent. of the whole; the two wet processes about 11 per cent., of which about two-thirds are contributed by the cyanide process; and, lastly, smelting produces 3 or 4 per cent. of the total.

All this is metallurgical, but the gold supply is intimately mixed up with many sociological questions, and no one who studies economics can doubt the great influence exerted on the happiness of the human race by any material oscillations in the production of a metal which is so universally accepted as a standard of value and a medium of exchange for the products of human labour.

